# Bicentric analysis of repeated retrosigmoid approach for recurrent vestibular schwannoma: facial nerve function and risk of second recurrence

**DOI:** 10.1007/s11060-026-05535-1

**Published:** 2026-03-24

**Authors:** Johannes Wach, Lisa Haddad, Martin Vychopen, Felix Arlt, Erdem Güresir, Katarzyna Rylewicz, Łukasz Przepiórka, Przemysław Kunert

**Affiliations:** 1 Department of Neurosurgery, University of Leipzig Medical Center, Liebigstraße 20, 04103 Leipzig, Germany; 2 Comprehensive Cancer Center Central Germany, Partner Site Leipzig, 04103 Leipzig, Germany; 3 Department of Neurosurgery, Medical University of Warsaw, Warsaw, Poland

**Keywords:** Facial nerve, Recurrence, Retrosigmoid craniotomy, Surgery, Vestibular schwannoma

## Abstract

**Background:**

Surgical retreatment of recurrent vestibular schwannoma (VS) remains challenging, particularly in balancing tumor control with facial nerve (FN) preservation. This bicentric study aimed to evaluate facial nerve outcomes and risk factors for second recurrence following repeated retrosigmoid craniotomy.

**Methods:**

We retrospectively analyzed 35 patients with sporadic VS who underwent repeated retrosigmoid surgery for recurrent VS between 2002 and 2024. Facial nerve function was assessed using the House-Brackmann (HB) grading. Predictors of postoperative FN deterioration at 3-months after repeated surgery and second recurrence were evaluated.

**Results:**

FN deterioration occurred in 11 patients (31.4%) at 3-months after repeated surgery. The time between primary and repeated surgery was significantly shorter in patients with postoperative FN deterioration (mean 42.4 vs. 81.2 months, *p* = 0.008). ROC analysis identified a cut-off of 102 months (AUC = 0.61; 95% CI: 0.42–0.79) for predicting facial nerve preservation. Regarding tumor control, a second recurrence was observed in 4 patients (11.4%). The volumetric extent of resection (EoR) during repeated surgery was significantly associated with second recurrence (*p* = 0.011). ROC analysis revealed a critical EoR cut-off of 62.0% (AUC = 0.96; 95% CI: 0.89–1.00), with 100% sensitivity and 92.9% specificity. Adjuvant radiotherapy after incomplete resection was not significantly associated with reduced re-recurrence (*p* = 0.77).

**Conclusions:**

Repeated retrosigmoid surgery for recurrent VS offers favorable tumor control with acceptable facial nerve outcomes. More aggressive VSs with shorter intervals between surgeries may increase the risk of facial nerve deterioration. Local repeated surgery appears to be of importance regarding further tumor control.

**Graphical Abstract:**

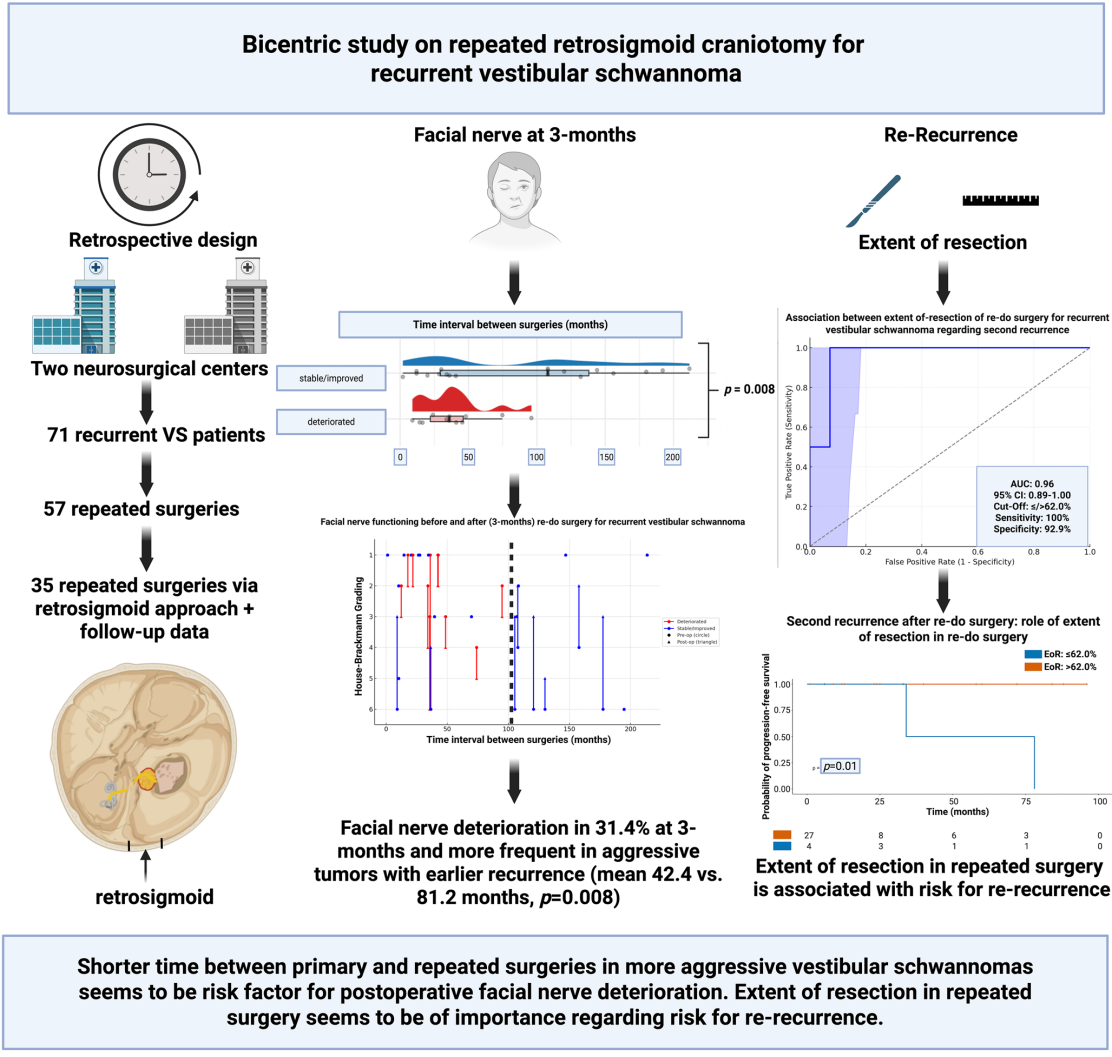

**Supplementary Information:**

The online version contains supplementary material available at 10.1007/s11060-026-05535-1.

## Introduction

Vestibular schwannomas (VSs) are benign, typically slow-growing tumors arising from the Schwann cells of the vestibulocochlear nerve [1]. While observation and stereotactic radiosurgery have become accepted first-line strategies, surgical resection remains necessary for large or symptomatic tumors causing brainstem compression, hydrocephalus, or cranial neuropathy [2]. Despite advances in microsurgical techniques, recurrence or progression following incomplete resection occurs in up to 20% of patients, often necessitating additional treatment [3]. 

The management of recurrent VS after initial resection poses a clinical challenge. While radiosurgery is frequently used in this setting, repeat microsurgery may be required in cases of significant mass effect or failure of prior treatments. However, literature on repeat VS surgery remains scarce, with limited data available on volumetric outcomes, functional preservation, and predictive factors for neurological deterioration. In one institutional series, only 6 of 102 patients (5.9%) required repeat resection, underlining the rarity of this scenario [4]. 

Furthermore, facial nerve (FN) function—a key outcome in VS surgery—is at greater risk during re-resection due to scarring and altered anatomy. (Nevertheless, evidence suggests that favorable outcomes remain achievable, particularly with the retrosigmoid approach and intraoperative neuromonitoring [5]. 

Given the paucity of detailed outcome data, this bicentric retrospective study aims to characterize FN outcome and local tumor control after re-resection via the retrosigmoid approach in patients with recurrent VS.

## Methods

### Study design

Between January 2002 and December 2024, 71 patients were treated for recurrent VSs.

Patients were eligible if they had previously undergone surgery via retrosigmoid approach for a VS, with or without radiation therapy, and had histopathologic confirmation of VS from both initial and repeat surgeries. Radiological evidence of tumor regrowth after either incomplete or complete resection was required, based on gadolinium-enhanced magnetic resonance imaging (MRI). All patients underwent repeat surgery via the retrosigmoid approach, with facial nerve function evaluated three months postoperatively. Patients who were treated for a recurrent VS via the translabyrinthine approach, underwent radiotherapy and with missing clinical follow-up were excluded. Patients with neurofibromatosis type 2 were not included due to the distinct biological behavior of their tumors.

### Surgical technique and imaging follow-up

A retrosigmoid craniotomy was performed to expose the junction of the transverse and sigmoid sinuses. After a curved dural incision, cerebrospinal fluid was gradually released from the cerebellomedullary cistern to expose the tumor. Intraoperative EMG monitoring was used in all cases, with recordings from the orbicularis oculi and oris muscles to assess FN function. Bipolar stimulation ranged from 1 to 0.05 mA with a pulse duration of 0.1 ms [6]. Center A performed the repeated surgeries for recurrent VS in supine lateral positioning, whereas Center B performed all surgeries in the semi-sitting positioning. First postoperative T1-weighted gadolinium-enhanced MR images after repeated surgery for recurrent VS is obtained at 3-months, and afterwards on an annual base.

### Data recording

The following general preoperative patient characteristics were documented and entered into a computerized database (SPSS, Version 29 for Windows, IBM Corp., Armonk, NY, USA): age, sex, time between primary and repeated surgery, facial nerve functioning before repeated surgery and at 3-months after repeated surgery, neurological deficits, and postoperative follow-up data. The extent of resection (EoR) was quantitatively assessed using volumetric measurements of tumor portions on T1-weighted gadolinium-enhanced MR images, calculated as: (preoperative tumor volume − postoperative tumor volume) / preoperative tumor volume [7]. Additionally, EoR was categorized our volumetric EOR into GTR (100%), NTR (> 95%), STR (90–95%), and PR (< 90%) [3]. Volumetric parameters were determined using Brainlab Smartbrush (BrainLAB AG, Feldkirchen, Germany). Complete resection was defined as an imaging finding without any residual nodular enhancement based on volumetry. Incomplete resection is any residual nodular enhancement determined in the volumetric analysis.

### Endpoints

Primary endpoint was the facial nerve functioning graded according to the House-Brackmann grading system [8]. Follow-up at 3-months after repeated surgery defined two outcome groups: the stable or improvement group (HB grades 1–2 or stable grades) and deterioration group (≥ 1-grade decline or remaining in grades 3–6). Tumor progression was defined as an increase in tumor volume of more than 20% compared to the previous MRI, accompanied by a clinical decision to initiate treatment [1–10]. 

### Statistical analysis

Statistical analyses were performed using R (version 4.3.1). Data distribution was assessed with the Kolmogorov–Smirnov test. Continuous variables are presented as mean ± SD or median (IQR), and categorical data as counts and percentages. Group comparisons used Fisher’s exact test for categorical and t-tests or Mann–Whitney U tests for continuous variables, as appropriate.

Receiver operating characteristic (ROC) curves were used assessed the predictive value of continuous variables (e.g., time to recurrence, tumor volume, extent of resection) for postoperative facial nerve outcome at 3-months after repeated surgery and re-recurrence, respectively. Cut-offs were defined by Youden’s index. Kaplan–Meier curves were used to analyze progression-free survival. A *p*-value < 0.05 was considered statistically significant.

## Results

### Study cohort

**Fig. 1:**
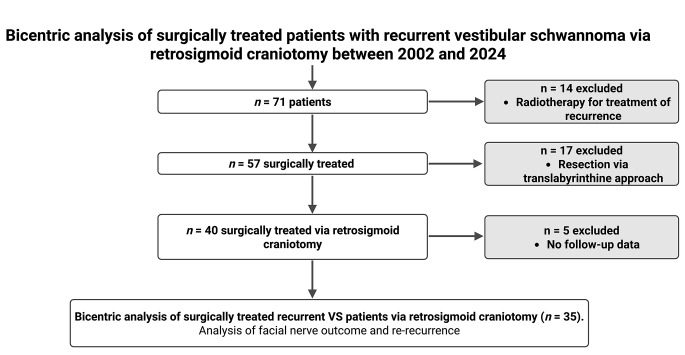
Flow diagram of patient selection. Between 2002 and 2024, 71 patients with recurrent vestibular schwannoma (VS) were treated. After exclusion of patients treated with radiotherapy alone (*n* = 14), translabyrinthine resection (*n* = 17), and incomplete follow-up data (*n* = 5), 35 patients who underwent re-resection via the retrosigmoid approach were included in the final analysis

Between 2002 and 2024, a total of 71 patients were treated for recurrent VS at two tertiary neurosurgical centers. Of these, 14 patients (19.7%) received radiotherapy only for the recurrence and were excluded. Among the remaining 57 surgically treated patients, 17 (29.8%) underwent resection via the translabyrinthine approach and were excluded. Five additional patients lacked follow-up data, resulting in a final study cohort of 35 patients who underwent repeated surgery via retrosigmoid craniotomy for recurrent VS and were included in the outcome analysis (Fig. 1).

### Patient characteristics

**Fig. 2:**
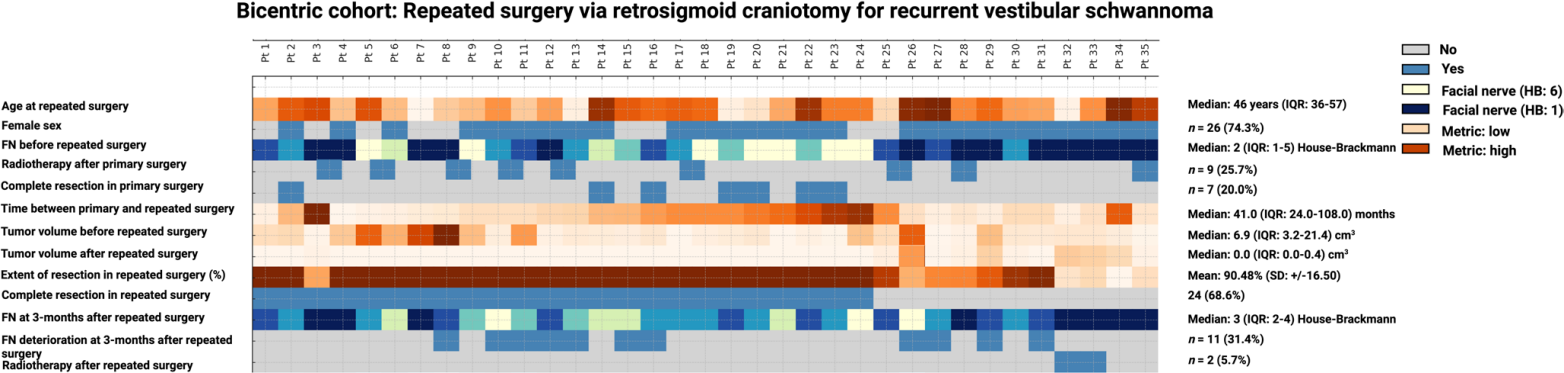
Oncoprint visualization of patient characteristics. Each column represents one of the 35 included patients. Rows depict demographic and clinical parameters including age, sex, facial nerve function (House-Brackmann grade) pre- and postoperatively, radiotherapy after primary or redo surgery, extent of resection (EoR), tumor volumes, and facial nerve outcome at 3 months. Color scales denote categorical (grey/blue) or continuous (yellow to red) variables as appropriate

The median age at repeated surgery was 46 years (IQR: 36–57), and 26 patients (74.3%) were female. Facial nerve (FN) function prior to repeated surgery was graded with a median House-Brackmann (HB) score of 2 (IQR: 1–5), with 11 patients (31.4%) presenting with severe dysfunction (HB ≥ 4) (Fig. 2). Radiotherapy after primary surgery was administered to 9 patients (25.7%), and 7 patients (20.0%) underwent complete resection at the initial surgery. The median time interval between primary and repeated surgery was 41.0 months (IQR: 24.0–108.0), and the median preoperative tumor volume before repeated surgery was 6.9 cm³ (IQR: 3.2–21.4 cm³). Further details are summarized in supplementary Table 1.

### Surgical outcomes

Following repeated surgery, the mean extent of resection (EoR) was 90.48% (SD+/- 16.50), and 24 patients (68.6%) achieved volumetrically confirmed complete resection (Fig. 2). The median residual tumor volume after repeated surgery for recurrent VS was 0.0 cm³ (IQR: 0.0–0.4 cm³). Median postoperative FN function at 3 months was HB grade 3 (IQR: 2–4). A total of 11 patients (31.4%) experienced deterioration of FN function, while 24 (68.6%) had stable or improved function. Only 2 patients (5.7%) with incompletely resected recurrent VS underwent radiotherapy after repeated surgery.

### Predictors of facial nerve deterioration

**Fig. 3:**
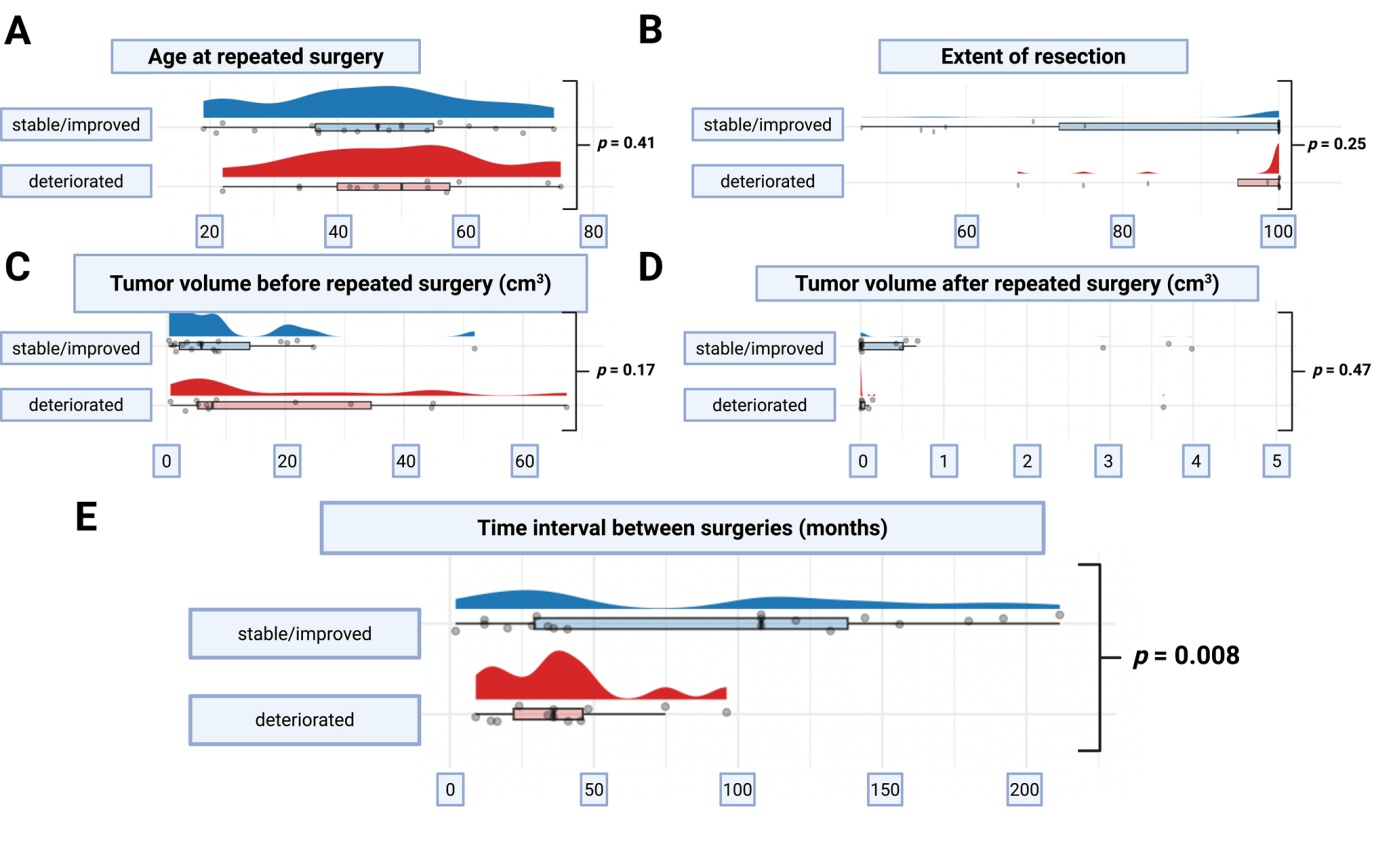
Comparison of clinical and radiological factors by facial nerve outcome. Raincloud plots showing (**A**) age at redo surgery, (**B**) extent of resection (%), (**C**) preoperative tumor volume, (**D**) postoperative tumor volume, and (**E**) time between primary and redo surgery. Patients with stable/improved facial nerve function are shown in blue; those with deterioration are shown in orange. Only the time interval between surgeries was significantly associated with facial nerve deterioration (*p* = 0.008)

Patients with postoperative FN deterioration were slightly older (median 50 years) compared to those with stable/improved outcomes (median 44 years, *p* = 0.41) (Fig. 3A). Although higher tumor volume before repeated surgery was observed in the deterioration group (median 9.4 vs. 5.5 cm³), this did not reach statistical significance (*p* = 0.17) (Fig. 3C). Likewise, extent of resection (*p* = 0.25) and postoperative residual tumor volume (*p* = 0.47) were not significantly associated with FN outcome (Fig. 3B, D). However, a significantly shorter time interval between surgeries was identified as a predictor of postoperative FN deterioration (median 27 vs. 61 months, *p* = 0.008) (Fig. 3E). Subgroup analysis by surgical positioning suggested a non-significant trend toward better facial nerve preservation in the lateral position. Among patients with stable or improved function, 75.0% were operated laterally versus 50.0% in the semi-sitting position. FN deterioration was more common in the semi-sitting group (54.5%) compared to the lateral group (21.7%), though this difference did not reach statistical significance (*p* = 0.13).

**Fig. 4:**
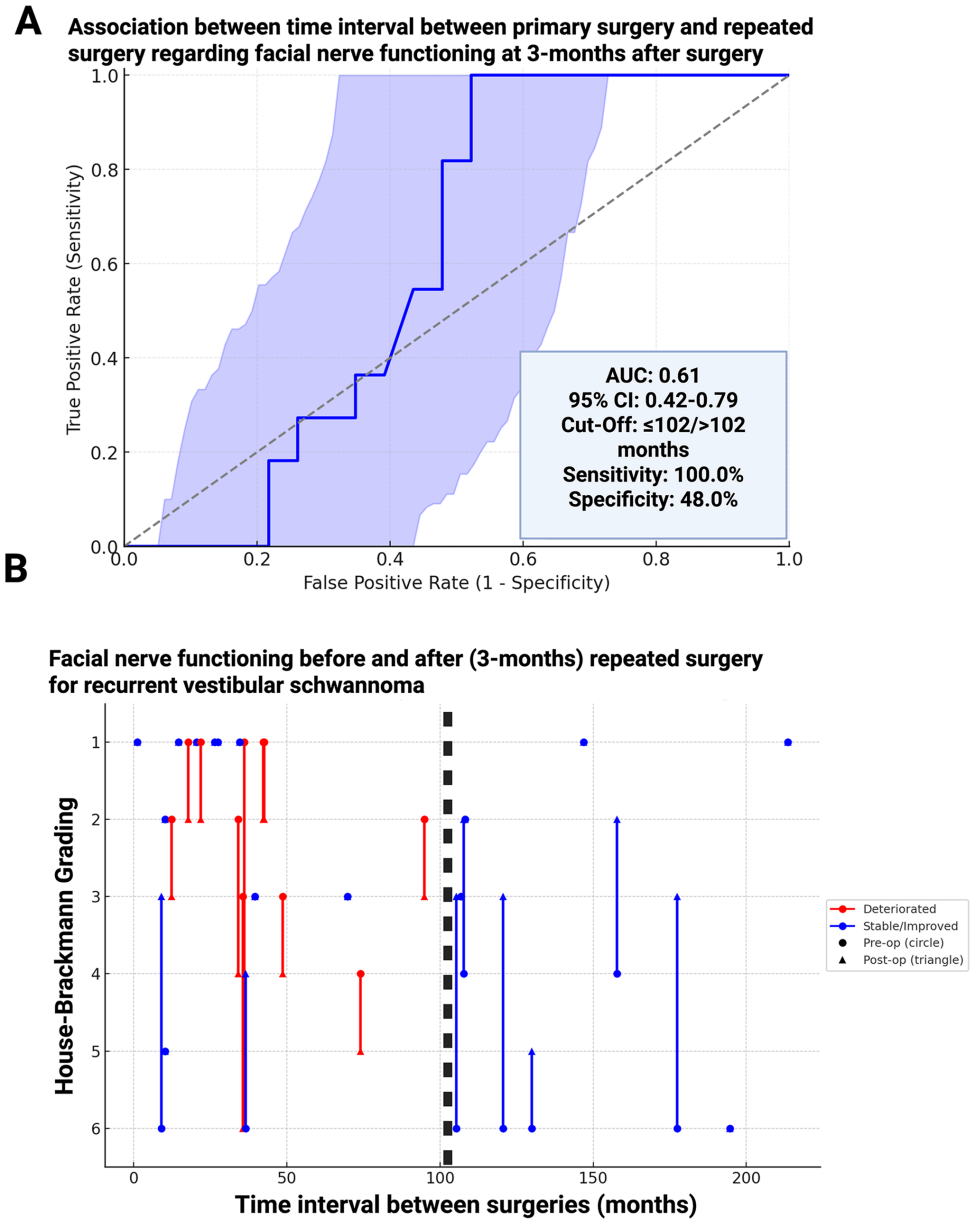
Receiver-operating characteristic (ROC) analysis and distribution of time intervals between primary and repeated surgery. (**A**) ROC curve showing predictive performance of time between surgeries for postoperative facial nerve deterioration at 3-months after repeated surgery. The optimal cutoff was ≤ 102 months (AUC: 0.61). (**B**) Trajectory plot showing individual patients and their facial nerve outcome at 3-months after repeated surgery for recurrent VS. Red trajectories indicate those with deterioration and being on the left side of the black dashed line indicating the cut-off for earlier and more aggressive VSs with higher risk for facial nerve deterioration

This association was further investigated in ROC curve analysis, which showed an AUC of 0.61 (95% CI: 0.42–0.79) for the time interval in predicting FN deterioration. An optimal cutoff of ≤ 102 months yielded 100% sensitivity and 48% specificity (Fig. 4A). As shown in Fig. 4B, FN deterioration primarily affected patients with shorter intervals between surgeries.

In the subgroup analysis of recurrent VS patients undergoing repeated resection without prior radiotherapy, group statistics showed comparable age at redo surgery (stable/improved: *n* = 17, mean 48.0 ± 15.5; deteriorated: *n* = 9, mean 47.9 ± 17.8), consistent with the non-significant comparison (*p* = 0.98). Extent of resection was similarly balanced (stable/improved: 89.0 ± 19.0 vs. deteriorated: 91.5 ± 13.1; *p* = 0.73). Tumor volumes did not differ significantly, although deteriorated cases trended higher: Preoperative recurrence volume 10.6 cm^3^ ± 10.6 vs. 21.2 cm^3^ ± 22.7 (*p* = 0.21) and postoperative volume 0.73 cm^3^ ± 1.38 cm^3^ vs. 2.10 ± 5.00 (*p* = 0.44). In contrast, the interval from primary surgery to recurrence was significantly longer in the stable/improved group (85.3 ± 65.5 months) than in deteriorated cases (39.5 ± 24.4 months; *p* = 0.0175), suggesting timing/disease progress may relate to short-term facial nerve outcome (see supplementary Fig. 1).

### Predictors of second recurrence

**Fig. 5:**
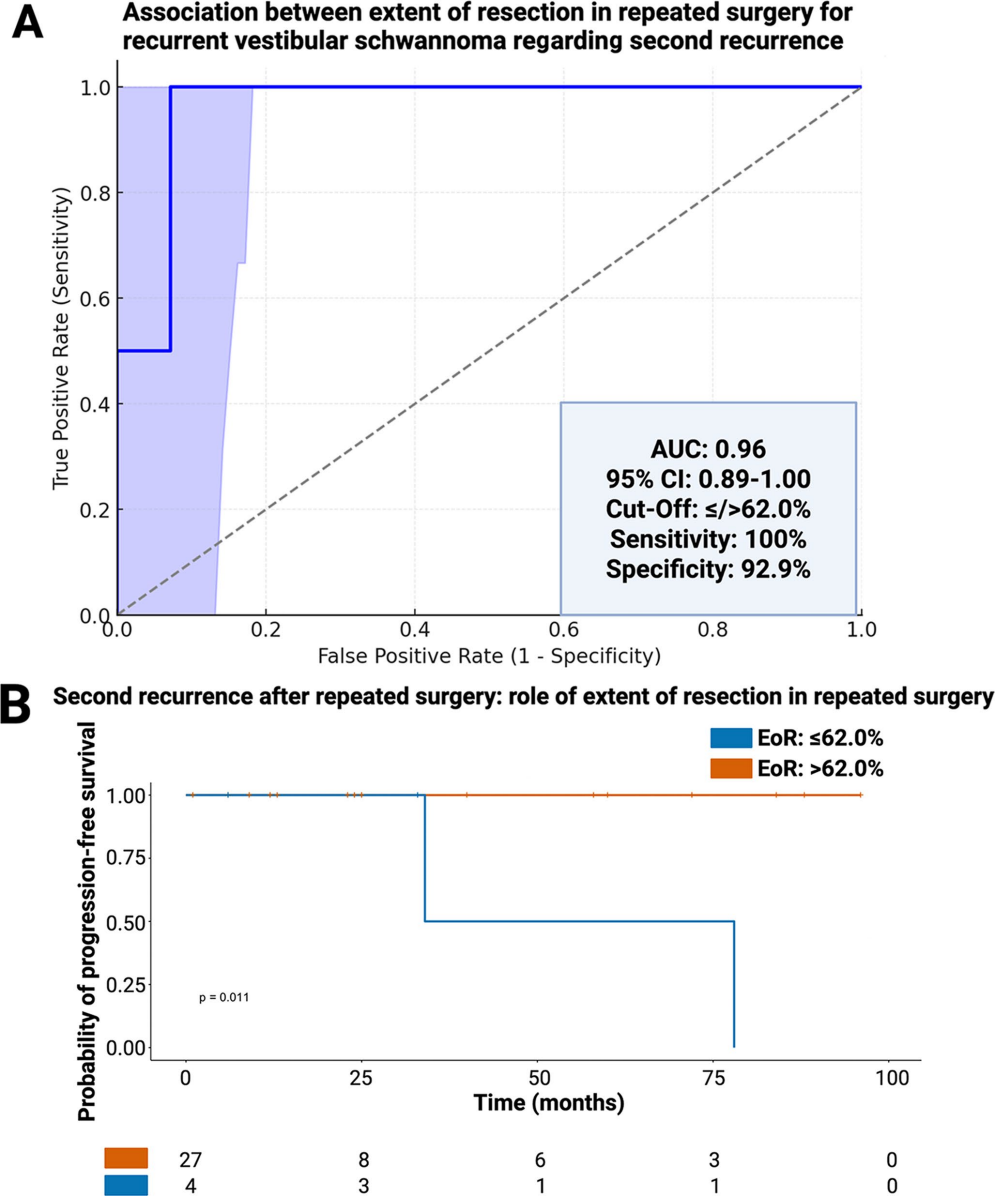

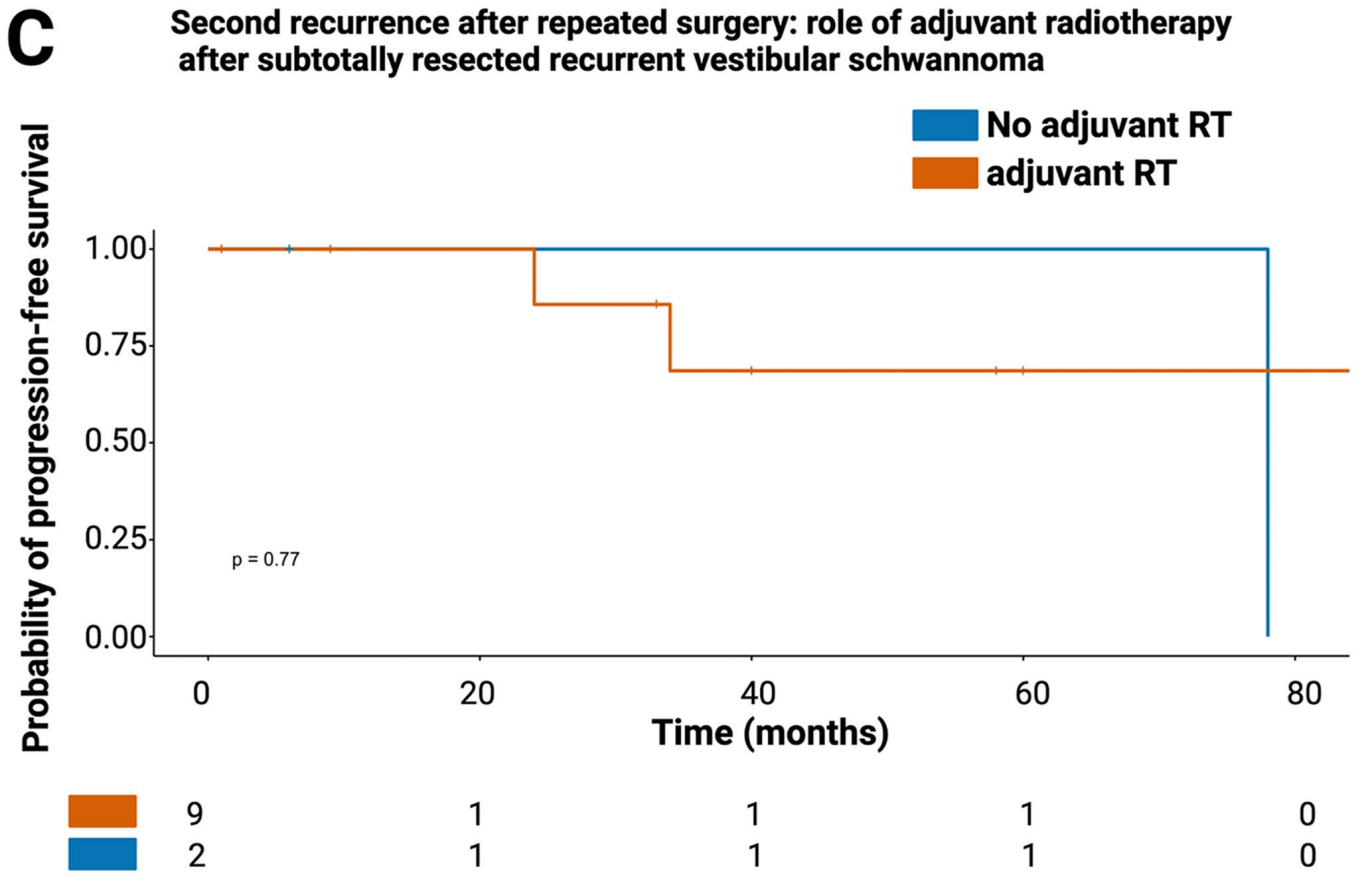
Extent of resection (EoR) and risk of second recurrence. (**A**) ROC curve demonstrating the predictive value of EoR for second recurrence (AUC: 0.96; cutoff ≤ 62.0%). (**B**) Kaplan–Meier progression-free survival curves stratified by EoR (≤ 62.0% vs. >62.0%, *p* = 0.011) and corresponding number at risk table. (**C**) Kaplan–Meier curves for progression-free survival in patients with subtotal resection, comparing those with vs. without adjuvant radiotherapy (*p* = 0.77)

Median MRI follow-up time after repeated surgery for recurrent VSs was 24 months (3–94 months). Second recurrence was observed in 4 out of 35 patients (11.4%). All second recurrences occurred in patients with an EoR ≤ 62.0% in repeated surgery for recurrent VS, while none were reported among patients with greater resection. ROC curve analysis revealed an AUC of 0.96 (95% CI: 0.89–1.00) for EoR in predicting a second recurrence, with a sensitivity of 100% and specificity of 92.9% at the ≤ 62.0% cutoff (Fig. 5A). Kaplan–Meier analysis confirmed that patients with EoR > 62.0% had significantly higher probability of progression-free survival regading re-recurrence (*p* = 0.011, Fig. 5B). In contrast, adjuvant radiotherapy after subtotal resection (*n* = 2) did not significantly impact progression-free survival (*p* = 0.77, Fig. 5C).

## Discussion

The management of recurrent VS remains a neurosurgical challenge. While primary surgical resection aims to achieve complete resection with maximal preservation of cranial nerve functioning, recurrence can still occur due to incomplete resection or tumor regrowth. The findings of the present bicentric study investigating repeated surgery for recurrent VS via the retrosigmoid approach can be summarized as follows: (1) More aggressive VSs with shorter time to recurrence may be associated with an increased risk for FN deterioration postoperatively. (2) Extent of resection in repeated surgery is of importance regarding local tumor control and development of re-recurrence.

The aim of repeated surgery for recurrent VS is predominantly oncological local tumor control and in large recurrent VSs decompression of the brainstem. These two cornerstones of the surgical treatment should be performed with preservation of the FN. Unlike initial VS surgery, a second procedure typically does not aim to preserve hearing, as most patients are already deaf on the affected side [11].

There are three therapeutic options for recurrent VS: observation, revision surgery, and radiosurgery. Both Tomita et al. [12] and Troude et al. [13] retrospectively investigated remnant tumors and suggested that small residual tumors can often be safely monitored through a conservative “wait and scan” strategy without immediate treatment. However, some patients experience a reduced quality of life due to anxiety from knowing about an untreated intracranial tumor, despite tumor stability [14]. repeated surgery, while definitively removing the tumor, potentially carries a high risk of complications in the setting of secondary surgery. Radiosurgery offers a less invasive approach but does not eliminate the tumor completely and is generally not suitable for larger lesions compromising the brainstem [15].

The current bicentric study analyzes FN outcomes highlighting both commonalities and contrasts. Our observed FN deterioration rate at 3-months of 31.4% postoperatively is within the range reported by Samii et al. [5], who retrospectively demonstrated tumor size and preoperative FN functioning as predictors for FN outcome. However, in this study patients were categorized into three categories of FN outcome and the individual delta of the FN functioning was not evaluated. Conversely, Perry et al. [16] retrospectively analyzed a case series of six patients who underwent repeat microsurgery for recurrent VS after a previously confirmed GTR. They reported that GTR was achieved in 67%, and House–Brackmann grade I–II facial nerve function was preserved in 83% of patients, with no significant postoperative worsening of FN. A key finding was that using an alternate surgical approach at the time of reoperation (in 67%) might mitigate scar-related challenges and facilitate safer dissection. In our study rates of complete resection increased from primary (20.0%) to repeated (68.8%) setting for recurrent VS, which underscores the different oncological attempt regarding EoR and local tumor control in this cohort of aggressive recurrent VSs. However, EoR in repeated surgery for recurrent VS did not emerge as a predictor of FN at 3-months after surgery. This finding is divergent to most of the data from treatment for primary VS because pooled datasets assume that subtotal or near-total resections are associated with better FN outcomes compared with gross total resection [17].

A notable finding in our study, which represents, to our knowledge, the largest series of repeated surgeries for recurrent VS using the retrosigmoid approach to date, is the association between shorter intervals between surgeries and an increased risk of facial nerve deterioration at the 3-month follow-up.

This may be explained by more aggressive tumor biology in rapidly recurring tumors or more complex scarring and tissue planes in early revisions. Previous studies have noted that early recurrence may correlate with proliferative indices or unfavorable histology, although our dataset did not include MIB-1 labeling or histomolecular subtyping. Several studies have linked higher MIB-1 indices to early tumor recurrence, suggesting a more aggressive biological behavior in these VSs [18, 19]. Panigrahi et al. [20] identified an MIB-1 threshold ≥ 3.5% as a strong predictor of regrowth in a retrospective investigation of 144 patients, while Charabi et al. [21] associated short symptom duration with elevated proliferative activity. In our study, patients with shorter intervals between initial surgery and recurrence exhibited higher rates of postoperative FN deterioration, possibly reflecting biologically active tumors with greater adhesiveness and inflammatory scarring. As inflammation-driven markers such as COX2, PD-L1, and tumor-associated macrophages co-localize with MIB-1, a proliferative signature may portend both recurrence and functional decline [22]. Furthermore, in a retrospective investigation of 118 primary sporadic VS patients treated via the retrosigmoid approach an increased MIB-1 labeling index was found to be associated with poor FN outcome [22]. Increased MIB-1 indices in VSs are associated with COX2 expression and suggest an inflammatory microenvironment promoting scar formation and tumor adhesion [23, 24]. This may explain the increased risk of facial nerve injury in patients with short recurrence intervals, as seen in our cohort. Rapidly recurring tumors likely reflect more aggressive and fibrotic biology, complicating resection.

In our bicentric cohort, EoR played a pivotal role in local tumor control after re-resection for recurrent VS. Re-recurrence is a rare event and was observed in 4 patients among the 35 recurrent VS patients. All second recurrences were observed in patients who had an EoR of ≤ 62.0% during repeated surgery for recurrent VS, whereas no such events occurred in those with a higher resection extent. This cut-off for EoR in repeated surgery yields a sensitivity and specificity of 100% and 92.9% regarding re-recurrence, respectively. To best of our knowledge, this finding represents the first data for the role of EoR in recurrent VS surgery and preservation of re-recurrent VS. However, this finding is consistent with previous studies investigating primary surgery for newly diagnosed VS, they reported a 3–12-fold increased risk of tumor regrowth and a threefold shorter median time to recurrence after subtotal resection compared to near-total resection [25–28]. The 62% EoR threshold underscores the importance of achieving a near-total resection during repeated surgery for recurrent VS when feasible. Surgeons may consider aiming for higher EoR to reduce recurrence risk, while carefully balancing the potential for FN injury.

### Limitations

Although this study represents the largest cohort of this rare condition to date, several limitations must be acknowledged. First, its retrospective design may introduce selection bias and restrict the ability to draw causal conclusions about factors influencing outcomes. Additionally, the bicentric setting could result in variability in surgical approaches, follow-up procedures, and imaging assessments, which may have affected the consistency of the results. However, the present investigation represents the largest cohort of surgically treated recurrent VS patients via the retrosigmoid approach. Another limitation is the lack of further long-term FN functioning even after repeated surgery. FN grading was assessed at 3 months postoperatively, and further longer-term functional trajectories remain unreported. Furthermore, as the study cohort only included patients deemed eligible for repeated surgery, there is a potential selection bias that may exclude more frail or surgically complex patients, potentially limiting the generalizability of the findings. Finally, despite being the largest series on repeated surgery via retrosigmoid craniotomy for recurrent VS, the cohort size of 35 patients does not allow a multivariable regression analysis of postoperative FN functioning. Additionally, future studies should address whether reoperation using a different surgical approach, such as the translabyrinthine approach, yields comparable or superior outcomes compared to re-do surgery via the same retrosigmoid approach, as our bicentric series lacked the statistical power and sufficient case numbers to allow for such an analysis.

## Conclusion

In conclusion, re-resection for recurrent VS via retrosigmoid craniotomy offers a viable treatment option for patients with radiological progression, especially when functional preservation is prioritized and radiosurgery is not suitable. Although facial nerve morbidity remains a risk, careful case selection and microsurgical technique can achieve satisfactory oncological and neurological outcomes. Near-total or total resection seems to be beneficial regarding the preservation of re-recurrence.

## Supplementary Information

Below is the link to the electronic supplementary material.

Supplementary Material 1

Supplementary Material 2: Figure 1. Subgroup analysis of recurrent vestibular schwannoma patients undergoing repeated resection without prior radiotherapy. Raincloud plots compare patients with stable/improved facial nerve function versus deteriorated facial nerve function at 3 months after repeated surgery (n = 17 vs n = 9, respectively). Panels depict (A) age at repeated surgery, (B) extent of resection (EOR, %), (C) tumor volume before repeated surgery (cm³), (D) tumor volume after repeated surgery (cm³), and (E) time interval between surgeries (months). Distributions are shown as half-violins (density), with overlaid boxplots (median and interquartile range) and individual patient values (dots). Blue indicates stable/improved outcomes and red indicates deteriorated outcomes. Reported p-values above brackets are from two-sided independent two-sample t-tests.

## Data Availability

The data sets generated and analyzed in the current study are available upon request from the corresponding author.
