# Supplementary material for: Bicentric analysis of repeated retrosigmoid approach for recurrent vestibular schwannoma: facial nerve function and risk of second recurrence
Source: J Neurooncol. 2026 Mar 24;177(2):73. doi: 10.1007/s11060-026-05535-1 (PMC13013203; doi:10.1007/s11060-026-05535-1)
Supplement: Supplementary file 1 — Supplementary Material 1 [file 11060_2026_5535_MOESM1_ESM.docx]

| **Supplementary Table 1. Patient Characteristics of the Study Cohort (*n* = 35)** | |
| --- | --- |
| **Variable** | **Value** |
| **Demographics** |  |
| Age at repeated Surgery (years) | 46.3 (36.0 – 57.0) |
| Female Sex | 26 (74.3%) |
|  |  |
| **Time Interval** |  |
| Time Between Primary and repeated Surgery (months) | 41.0 (24.0 – 108.0) |
| **Tumor Volume (cm³)** |  |
| Preoperative Tumor Volume Before repeated Surgery | 6.9 (3.2 – 21.4) |
| Postoperative Tumor Volume After repeated Surgery | 0.0 (0.0 – 0.4) |
|  |  |
| **Facial Nerve Function (House–Brackmann Grade)** |  |
| Preoperative Function Before repeated Surgery | 2.0 (1.0 – 5.0) |
| Postoperative Function at 3 Months After repeated Surgery | 3.0 (2.0 – 4.0) |
|  |  |
| **Surgical and Adjuvant Treatment Characteristics** |  |
| Adjuvant Radiotherapy After First Surgery | 9 (25.7%) |
| Adjuvant Radiotherapy After repeated Surgery | 2 (5.7%) |
| Complete Resection (GTR) in First Surgery | 7 (20.0%) |
| Semi-Sitting Patient Positioning in repeated Surgery | 12 (34.3%) |
| Complete Resection (GTR) in repeated Surgery | 24 (68.6%) |
| Extent of Resection (%) in repeated Surgery | 100 (77.2 – 100.0) |
