# Supplementary figures and images for: Bicentric analysis of repeated retrosigmoid approach for recurrent vestibular schwannoma: facial nerve function and risk of second recurrence
Source: J Neurooncol. 2026 Mar 24;177(2):73. doi: 10.1007/s11060-026-05535-1 (PMC13013203; doi:10.1007/s11060-026-05535-1)

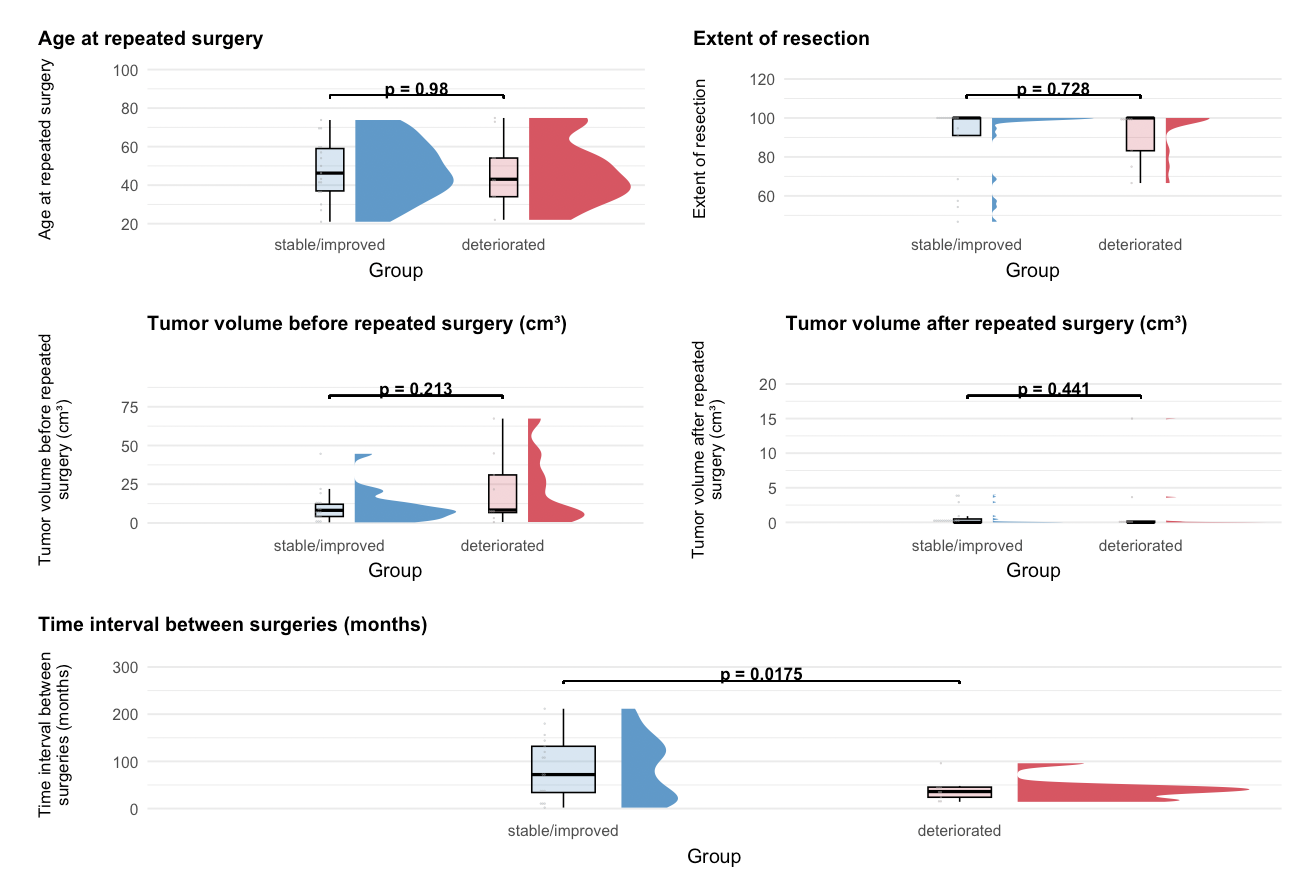

Supplement: Supplementary file 2 — Supplementary Material 2: Figure 1. Subgroup analysis of recurrent vestibular schwannoma patients undergoing repeated resection without prior radiotherapy. Raincloud plots compare patients with stable/improved facial nerve function versus deteriorated facial nerve function at 3 months after repeated surgery (n = 17 vs n = 9, respectively). Panels depict (A) age at repeated surgery, (B) extent of resection (EOR, %), (C) tumor volume before repeated surgery (cm³), (D) tumor volume after repeated surgery (cm³), and (E) time interval between surgeries (months). Distributions are shown as half-violins (density), with overlaid boxplots (median and interquartile range) and individual patient values (dots). Blue indicates stable/improved outcomes and red indicates deteriorated outcomes. Reported p-values above brackets are from two-sided independent two-sample t-tests. [file 11060_2026_5535_MOESM2_ESM.png]
